# Regeneration and Repair in Endodontics—A Special Issue of the *Dentistry Journal*

**DOI:** 10.3390/dj3030077

**Published:** 2015-08-19

**Authors:** Louis M. Lin, George T.-J. Huang

**Affiliations:** 1 Department of Endodontics, New York University College of Dentistry, 345 East 24th Street, New York, NY 10010, USA; 2 Director for Stem Cells and Regenerative Therapies, Department of Bioscience Research College of Dentistry, University of Tennessee Health Science Center, Cancer Research Building, 19 S. Manassas St., Lab Rm 225, Office Rm 222, Memphis, TN 38163, USA; E-Mail: gtjhuang@uthsc.edu

Endodontics is a specialized discipline in dentistry that concerns the morphology, physiology, and pathology of the pulp-dentin complex, root, and peri-radicular tissues. These tissues possess various regenerative potentials when damaged. The discovery of dental stem cells in the past decade has helped researchers in this field examine the regeneration and repair of these endodontic tissues from a new perspective. This Special Issue launches a comprehensive review covering the recent understanding of regeneration and repair of the dentin-pulp complex and the periapical tissues after injury, as well as the advancement in regenerative endodontic therapy. The following specific areas will be discussed: (i) cellular and molecular biology of wound healing concerning the mechanisms of regeneration and repair; (ii) the outcomes of various treatments of pulpal and periapical disease, including direct pulp capping, apexogenesis, apexification, root canal treatment and regenerative endodontic therapy in the context of regeneration and repair; (iii) the challenges and hopes of regeneration of the dentin-pulp complex in teeth with necrotic pulps; and (iv) recent advances in regenerative endodontic therapy of immature and mature teeth with infected or non-infected necrotic pulps.

Topics might include but are not limited to:

- Pulp-dentin complex;
- Apexogenesis;
- Apexifiction;
- Direct pulp capping;
- Dental stem cells;
- Regeneration;
- Regenerative endodontic therapy;
- Repair;
- Wound healing;
- Root canal therapy;
- Periapical tissues;
- Clinical outcome.

